# Supplementary material for: Sequence Evaluation and Comparative Analysis of Novel Assays for Intact Proviral HIV-1 DNA
Source: J Virol. 2021 Feb 24;95(6):e01986-20. doi: 10.1128/JVI.01986-20 (PMC8094944; doi:10.1128/JVI.01986-20)
Supplement: Supplemental file 1 [file JVI.01986-20-s0001.pdf]

Supplementary Tables Legend

Table S1: Participant Characteristics. Age, sex, race/ethnicity, time since HIV diagnosis, time since ART initiation, ART regimen at time of donation, and estimated CD4 nadir are provided for the n=39 ART-suppressed participants studied.

Table S2: Quantitative Analysis. Table S2 shows the results of the quantitative reservoirs measurements for the 39 ART-suppressed participants studied.

Table S1: Individual participant demographics and baseline clinical characteristics

ID	Age	Sex	Race	Years since HIV-1 dx	Years since first ART	ART at Screening	Estimated CD4 nadir
**9241**	40	M	White/Hisp	6	7	EVG/cobi/TDF/FTC	500
**9242**	43	M	White/Hisp	3	3	EVG/cobi/ TDF/FTC	450
**9243**	29	M	Amer Indian/Hisp	5	5	RPV/TDF/FTC	350
**9244**	36	M	Amer Indian/Non-Hisp	9	5	EFV/TDF/FTC	730
**9246**	30	M	Black	5	5	EVG/cobi/ TAF/FTC	500
**9247**	31	M	Black	6	6	EVG/cobi/ TAF/FTC	600
**9252**	51	F	Black	11	11	EFV/TDF/FTC	270
**9254**	48	M	White	21	21	EVG/cobi/ TAF/FTC	590
**9255**	30	M	White	5	4	EVG/cobi/ TAF/FTC	779
**B207**	48	M	White/Hisp	12	11	EVG/TDF/FTC	not available
**603**	45	M	White/Hisp	14	12	EFV/TDF/FTC	300
**605**	38	M	White/Hisp	17	16	RPV/TDF/FTC	372
**5104**	35	M	Black	7	7	BIC/TAF/FTC	400
**5106**	31	M	Black	6	6	EVG/COBI/FTC/TDF	>350
**5108***	52	M	White	10	10	EVG/COBI/FTC/TAF	>300
**5112***	30	M	White	6	5	EVG/COBI/FTC/TAF	350
**5101**	52	M	Multiple/Hisp	12	12	TDF/FTC/EFV	not available
**5114**	54	M	Black	15	15	ABC/DTG/3TC	300
**5105**	49	F	Black	29	29	FTC/RPV/TDF	200
**5203**	59	M	White	23	21	DTG/RPV	500
**5111**	55	M	White	20	16	EVG/COBI/FTC/TAF	400
**5115**	36	M	Black	6	6	FTC/TAF/DTG	not available
**TSC-124**	52	M	Black	10	10	EFV/TDF/FTC	not available
**TSC-127**	57	F	Multiple/Non-Hisp	20	20	EVG/COBI/FTC/TAF	not available
**TSC-125**	43	M	White	18	11	EVG/COBI/FTC/TDF	not available
**TSC-131**	45	M	Multiple	1	1	EFV/TDF/FTC	not available
**TSC-128**	43	M	N/A / Non-Hisp	18	18	FTC/RPV/TDF	not available
**UNC-432**	51	M	White	11	11	ABC/DTG/3TC	403
**UNC-308**	53	M	White	22	16	DRV/ABC/3TC/NEV/RTV	81
**UNC-346**	51	M	White	10	9	EVG/cobi/FTC/TAF	168
**UNC-367**	28	M	White	6	5	DRV/cobi/FTC/TAF	168
**UNC-412**	31	M	White	8	8	ABC/DTG/3TC	354
**UNC-434**	45	M	Other/Hisp	6	6	BIC/FTC/TAF	526
**UNC-404**	25	M	Black	2	2	ABC/DTG/3TC	308
**UNC-336**	53	M	Black	22	20	EVG/cobi/FTC/TAF	462
**UNC-406,425****	43/44	M	Black	1,2	1,2	ABC/DTG/3TC	713
**UNC-397**	34	M	Black	10	4	EVG/cobi/FTC/TAF	not available
**UNC-437**	42	F	Black	9	8	EVG/cobi/FTC/TAF	350
**UNC-458***	42	F	Black	11	11	BIC/FTC/TAF	not available

EVG - elvitegravir, cobi - cobicistat, TDF - tenofovir disoproxil fumarate, FTC - emtricitabine,
RTV - ritonavir, ABC - abacavir, 3TC - lamivudine, DTG - dolutegravir. BIC - bictegravir
RPV - rilpivirine, EFV - efavirenz, TAF - tenofovir alafenamide fumarate, DRV - darunavir, NEV- nevirapine
*These participants harbored non-clade B viruses.
**This individual provided 2 donations at 1 year and 2 years following ART initiation.

Table S2: Quantitative Analysis

ID	Intact proviruses per 10^6 $CD4^+$ T cells		IPDA env RRE-defective	IPDA PS-defective	IPDA Total	Q(2)VOA or QVOA (IUPM)	Total HIV DNA (gag)	DNA Shearing Index	Cell equivalents assayed (IPDA)	Cell equivalents assayed (Q4PCR)	Q4PCR PS+env per 106 cells (< 38 CT, non-sequence confirmed)	Genotype	Notes
	Q4PCR (PS+*env*+, sequence confirmed)	IPDA (PS+*env*+)											
**9243_wk-2**	3.47	amplification failure	amplification failure	amplification failure	amplification failure	0.17	413	34.9	1385822	2304000	10.42	B	PS amplification failure
**9243_wk12**	1.12	amplification failure	amplification failure	amplification failure	amplification failure	0.126	437	33.6	1031869	2688000	4.46	B	PS amplification failure
**9244_wk-2**	2.60	amplification failure	amplification failure	amplification failure	amplification failure	0.397	359	34.9	1430494	3840000	0.26	B	PS amplification failure
**9244_wk12**	2.60	amplification failure	amplification failure	amplification failure	amplification failure	0.354	346	35.2	1014435	2304000	0.00	B	PS amplification failure
**605**	0.919	amplification failure	amplification failure	amplification failure	amplification failure	0.6	294	38.5	1191036	6528000	0.00	B	PS amplification failure
**5108**	9.90	amplification failure	amplification failure	amplification failure	amplification failure	-	224	32.1	1061720	1920000	3.65	G	env amplification failure
**5112**	3.91	amplification failure	amplification failure	amplification failure	amplification failure	-	363	33.2	1283070	1536000	0.65	A1	env amplification failure
**5106**	7.29	amplification failure	amplification failure	amplification failure	amplification failure	-	22	32.2	1079568	1920000	34.90	B	env amplification failure
**9241**	6.25	amplification failure	amplification failure	amplification failure	amplification failure	0.743	696	33.4	1059815	960000	19.79	B	env amplification failure
**9242_wk-2**	9.90	367	650	517	1534	0.781	955	36.7	1118546	3840000	22.92	B	env polymorphsm preventing robust gating
**9242_wk12**	8.07	373	745	573	1691	0.493	1107	36.6	1266640	3840000	19.53	B	env polymorphsm preventing robust gating
**5101**	5.21	87	473	2045	2605	-	406	44.4	647294	768000	88.54	B	env polymorphsm preventing robust gating
**9252_wk-2**	8.52	64	379	260	703	1.709	449	35.2	1118546	2112000	1.42	B	env polymorphsm preventing robust gating
**9252_wk12**	3.04	59	368	242	669	1.47	389	34.2	1266640	2304000	3.47	B	env polymorphsm preventing robust gating
**5114**	4.17	59	570	610	1239	-	666	35.2	547882	960000	16.67	B	PS polymorphism preventing robust gating
**B207**	11.3	712	1153	1449	3314	13.0	3047	36.7	1021616	1152000	9.55	B	No gating issues
**5105**	3.47	45	376	493	914	-	604	32.1	794263	864000	13.89	B	No gating issues
**5203**	39.06	56	118	307	481	-	173	36	932879	384000	54.69	B	No gating issues
**9255_wk-2**	6.51	76	261	162	499	1.89	387	34.9	1267573	3072000	0.00	B	No gating issues
**9255_wk12**	7.49	66	232	231	529	1.4	414	44.3	483521	3072000	0.00	B	No gating issues
**603**	0.95	41	490	278	809	3.75	598	34.7	1264970	8448000	2.72	B	No gating issues
**5104**	215.28	233	288	214	735	-	546	30.1	1126208	576000	262.15	B	No gating issues
**UNC-308**	4.17	204	2088	785	3077	0.854	2676	33.1	1157749	480000	20.83	B	No gating issues
**UNC-346**	12.25	85	970	495	1550	1.485	868	32.2	1157347	652800	59.74	B	No gating issues
**UNC-367**	5.21	297	1049	649	1995	4.626	1235	36	857048	960000	39.58	B	No gating issues
**UNC-404**	0.37	328	88	61	477	0.816	277	35.4	1010500	2688000	2.98	B	No gating issues
**UNC-412**	0.74	22	218	257	497	0.553	190	34.5	550541	1344000	8.18	B	No gating issues
**UNC-406,425_wk48**	0	124	19	33	176	0.827	214	50.8	1379079	1804800	0.00	B	No gating issues
**UNC-406,425_wk96**	0.00	47	42	61	150	0.279	114	40.2	827052	2265600	3.09	B	No gating issues
**UNC-432**	1.13	31	977	135	1143	0.164	1168	36.8	835075	883200	64.54	B	No gating issues
**UNC-434**	4.69	203	424	194	821	0.558	772	41.3	950662	1920000	11.98	B	No gating issues
**UNC-458**	0.00	5	10	49	64	0.452	341	39.8	758396	652800	41.36	A1	No gating issues
**9246**	1.3	86	182	162	430	0.066	215	38.6	882542	768000	39.06	B	No gating issues
**9247**	27.08	7	32	48	87	0.0249	20	34.1	1016764	960000	31.25	B	No gating issues
**5111**	6.0	38	99	257	394	-	171	31.5	983221	1344000	6.70	B	No gating issues
**5115**	0	26	368	220	614	-	452	32.6	962607	5376000	1.30	B	No gating issues
**TSC 124**	0	5	18	33	56	-	20	32.9	728487	2496000	1.60	B	No gating issues
**TSC 127**	14.42	91	1045	1734	2870	-	1241	33.8	950293	624000	40.06	B	No gating issues
**TSC 125**	1.86	118	756	583	1457	-	724	33.1	929829	537600	5.58	B	No gating issues
**TSC 131**	0	20	53	23	96	-	amplification failure	32.2	1086317	768000	2.60	B	No gating issues
**TSC 128**	1.60	271	698	632	1601	-	187	27.8	584973	2496000	7.21	B	No gating issues
**UNC-336**	0	10	133	189	332	0.021	153	33.7	829956	1574400	0.00	-	No gating issues
**UNC-397**	0	5	62	9	76	0.021	87	35.5	1620390	1881600	0.00	-	No gating issues
**UNC-437**	0	108	357	170	635	0.604	amplification failure	35.1	971848	1344000	20.09	B	No gating issues
**9254**	27.34	40	242	85	367	-	172	34.2	808535	1536000	33.85	B	No gating issues
